# Antiproliferative Effect of Aminoethyl-Chitooligosaccharide on Human Lung A549 Cancer Cells

**DOI:** 10.3390/biom9050195

**Published:** 2019-05-18

**Authors:** Dai Hung Ngo, Dai Nghiep Ngo, Se-Kwon Kim, Thanh Sang Vo

**Affiliations:** 1 Faculty of Natural Sciences, Thu Dau Mot University, Thu Dau Mot City 820000, Binh Duong province, Vietnam; hungnd@tdmu.edu.vn; 2 Department of Biochemistry, Faculty of Biology and Biotechnology, University of Science, Vietnam National University, Ho Chi Minh City 700000, Vietnam; ndnghiep@hcmus.edu.vn; 3 Department of Marine Life Science, College of Ocean Science and Technology, Korea Maritime and Ocean University, Busan 606-791, South Korea; sknkim@pknu.ac.kr; 4 NTT Hi-Tech Institute, Nguyen Tat Thanh University, Ho Chi Minh City, Vietnam; vtsang@ntt.edu.vn

**Keywords:** chitooligosaccharide, proliferation, apoptosis, A549 cells, caspase-3

## Abstract

The aminoethyl–chitooligosaccharide (AE-COS) was reported to inhibit human gastric cancer cell proliferation and human fibrosarcoma cell invasion. In this study, the role of AE-COS in down-regulation of proliferation of human lung A549 cancer cells was evaluated. It was found that AE-COS was able to reduce A549 cell proliferation to (32 ± 1.3)% at a concentration of 500 µg/mL. Moreover, AE-COS treatment caused suppression on COX-2 expression in a dose-dependent manner. Notably, the role of AE-COS in induction of cell apoptosis was observed via decreasing Bcl-2 expression and increasing caspase-3 and -9 activation. Accordingly, the antiproliferative effect of AE-COS was indicated due to suppression of cell proliferation and induction of cell apoptosis, suggesting AE-COS as a promising chemotherapy agent for treatment of lung cancer.

## 1. Introduction

Cancers are well known for their involvement in unregulated cell growth, proliferation, invasion, and metastasis [1]. Among them, lung cancer is one of the most leading causes of morbidity and mortality worldwide [2]. The difficulty of early diagnosis as well as high potential of invasion and metastasis have resulted in the high mortality rates of lung cancer. Lung cancer originates from normal lung epithelial cells which transform into uncontrolled proliferating cells in the lung airway. Two major types of lung cancer have been classified as nonsmall cell lung cancer and small-cell lung cancer that facilitate the prediction and treatment strategy. Notably, the important feature of cancer cells was identified due to evading apoptosis [3]. Thus, induction of apoptosis is considered as a potential strategy for cancer treatment. 

Chitin, a unique polysaccharide, consists of an *N*-acetylglucosamine unit and makes up the exoskeletons of crustaceans and invertebrates. The deacetylation of chitin up to 50% could lead to generation of aqueous acidic soluble form, called chitosan. Meanwhile, the hydrolysis of chitosan forms chitooligosaccharides (COS), which were evidenced to possess biodegradable, nontoxic, and non-allergenic properties and numerous biological activities [4,5]. Thus, COS has the potential for application in various fields, such as agriculture, biomedicine, food, cosmetics, and the environment [6]. Recently, numerous COS derivatives have been produced via introducing the bioactive group onto natural COS backbone to increase its biological activities as well as availability [7]. According to Rajapakse and colleagues, carboxylated chitooligosaccharides (CCOS) were synthesized by adding a carboxyl group to the amino position of pyranose unit [8]. CCOS were determined to possess inhibitory activities on MMP-9, angiotensin-converting enzyme, and ROS production [9,10]. Ngo et al. [11] synthesized gallic acid-conjugated COS (G-COS). Its biological activities were found due to antioxidant [12] and anti-allergic effects [13]. Moreover, sulfated COS (COS-S) exhibited antioxidant [14], anti-HIV [15], and collagenases 1 and 3 suppression [16]. Especially the combination of COS and the aminoethyl group gains an interest in the inhibition of human fibrosarcoma cell invasion, which may contribute to the prevention of metastatic cancer cells [17]. In this study, aminoethyl–chitooligosaccharide (AE-COS) was evaluated for its anticancer activity due to inhibiting proliferation and inducing apoptosis in human lung A549 cancer cells.

## 2. Materials and Methods 

### 2.1. Materials

A549 cells were bought from Korea Cell Line Bank (Seoul, Korea). AE-COS was provided by Dr. Dai Nghiep Ngo (Vietnam National University, Ho Chi Minh City, Vietnam). Enzyme immunoassay reagents were purchased from R&D Systems (Minneapolis, MN, USA). Antibodies were obtained from Santa Cruz Biotechnology Inc. (Santa Cruz, CA, USA). All other reagents were obtained from Sigma–Aldrich (St. Louis, MO, USA).

### 2.2. Aminoethyl–Chitooligosaccharides (AE-COS)

**Scheme 1:**
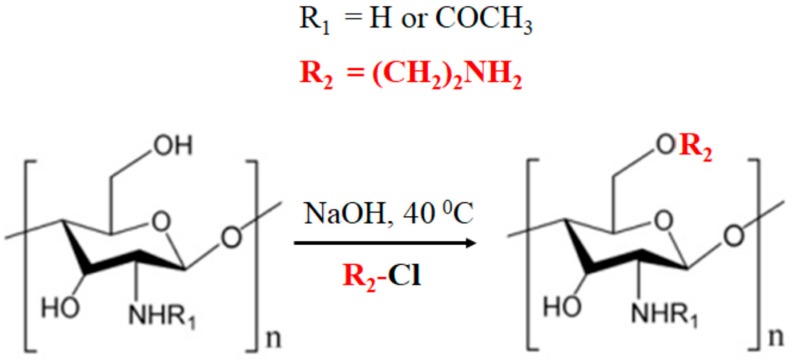
Synthesis of aminoethyl–chitooligosaccharides (AE-COS).

AE-COS (Scheme 1) was provided by Dr. Dai Nghiep Ngo (Vietnam National University, Ho Chi Minh City). The synthesis was conducted as described by Ngo and colleagues [18]. AE-COS were characterized by a ^1^HNMR (JEOL JNM ECP-400 NMR spectrometer under a static magnetic field of 400 MHz, JEOL, Tokyo, Japan) and MALDI-TOF mass spectrometry (Voyager mass spectrometer, Applied Biosystems, Waltham, Massachusetts, USA). Herein, the hydroxyl group at C-6 was successfully replaced by the aminoethyl group on pyranose ring structure. AE-COS has a molecular weight of 800.79–4765 Da, while COS has a molecular weight of 800–3000 Da and deacetylation degree of 90%.

### 2.3. Cell Viability Assay

The cytotoxic effect of AE-COS on A549 cells was determined by MTT (3-(4,5-Dimethylthiazol-2-yl)-2,5-Diphenyltetrazolium Bromide) assay. A549 cells (2 × 10^5^ cell/mL) were treated with AE-COS (100, 200, or 500 µg/mL) for 24 h before being incubated with MTT solution (1 mg/mL, final concentration) for 4 h. The supernatant was then removed, and DMSO (100 µL) was added to solubilize the formed formazan salt. The absorbance was measured at 540 nm using a microplate reader (GENios^®^ Tecan Austria GmbH, A-5082 Grödig/Salzburg, Austria). The cell viability was shown as a percentage compared to blank. Moreover, the morphology of AE-COS-treated A549 cells was observed under a light microscope (Olympus Corporation, Tokyo, Japan).

### 2.4. Western Blot Analysis

The protein expression level was measured by Western blot assay. Various doses of AE-COS (100, 200, or 500 µg/mL) were introduced to A549 cells for 24 h. The procedure was performed as described by Vo and Kim [19]. The protein band was visualized using LAS3000^®^ Luminescent image analyzer (Fujifilm Life Science, Tokyo, Japan).

### 2.5. Statistical Analysis

Data were analyzed using the analysis of variance (ANOVA) test of statistical package for the social sciences (SPSS Inc., Chicago, IL, USA). The statistical differences among groups were assessed using Duncan’s multiple range tests. Differences were considered significant at *p* < 0.05.

## 3. Results and Discussion

### 3.1. The Inhibitory Effect of AE-COS on A549 Cell Proliferation

**Figure 1:**
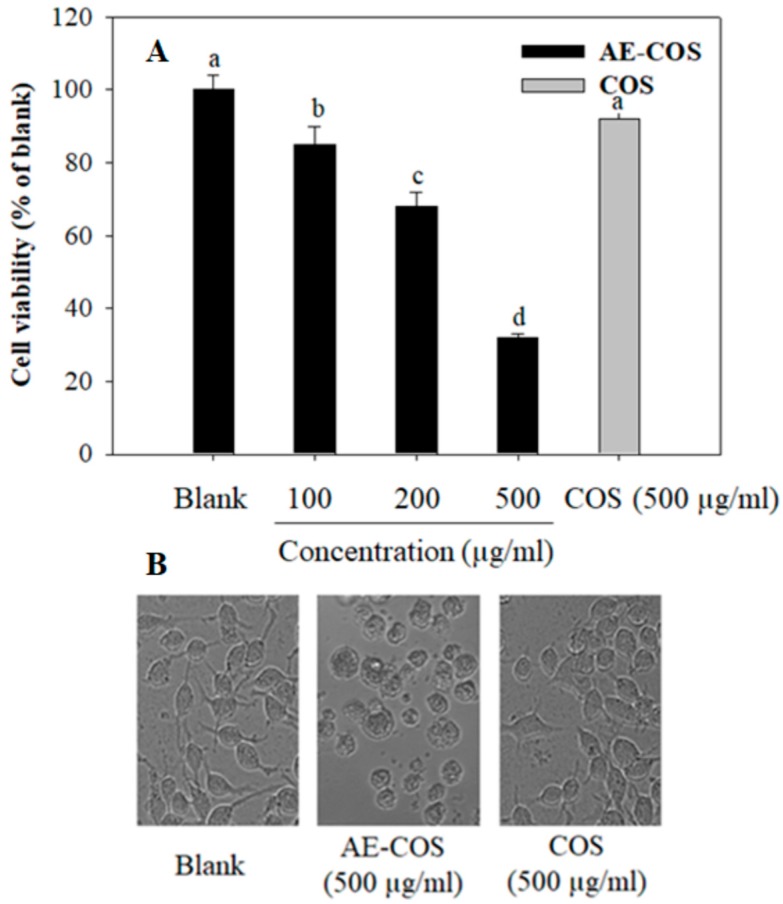
The inhibitory effect of AE-COS on A549 cell proliferation. The cells were treated with various concentrations of AE-COS for 24 h. (**A**) Cell viability was assessed via the MTT method. (**B**) The representative images of the cells were assessed by light microscopy (magnification, × 10). Each determination was made in triplicate, and the data were expressed as means ± SD. Different letters a–d indicate significant difference among groups (*p* < 0.05). Blank: No addition of AE-COS.

Cancer evolution and progression are involved in the unregulated cell proliferation and suppression of apoptosis [20]. Thus, the important parameter for screening anticancer agents is due to suppressing cancer cell proliferation and inducing cell apoptosis. In this assay, the inhibitory effect of AE-COS on the proliferation of human lung A549 cancer cells was investigated. The cells were treated with different concentrations of AE-COS for 24 h, and cell viability was then measured via MTT assay. The result showed that AE-COS treatment significantly reduced cell viability in a dose-dependent manner (Figure 1A). Especially, cell viability was reduced to (32 ± 1.3)% upon treatment of 500 µg/mL of AE-COS. Moreover, the inverted microscopy method showed that morphological changes occurred in AE-COS-treated cells (Figure 1B). The cells were observed to be round-shaped, with a reduced cell size, disrupted boundaries, and irregular surfaces, indicating cell injury and death. Meanwhile, COS did not cause any change on cell viability as well as cell morphology significantly. This indicated that the grafting of the aminoethyl group onto COS enhances its inhibitory activity on cell proliferation and causes cell injury and death. Ngo et al. [18] showed that AE-COS is more effective than COS in inhibition of angiotensin-converting enzyme. Likewise, Karagozlu et al. [21] revealed that COS derivatives such as aminoethyl COS, dimethyl aminoethyl COS, and diethyl aminoethyl COS decreased cell viability and induced cell apoptosis in AGS cells. 

### 3.2. The Suppressive Effect of AE-COS on COX-2 Expression

**Figure 2:**
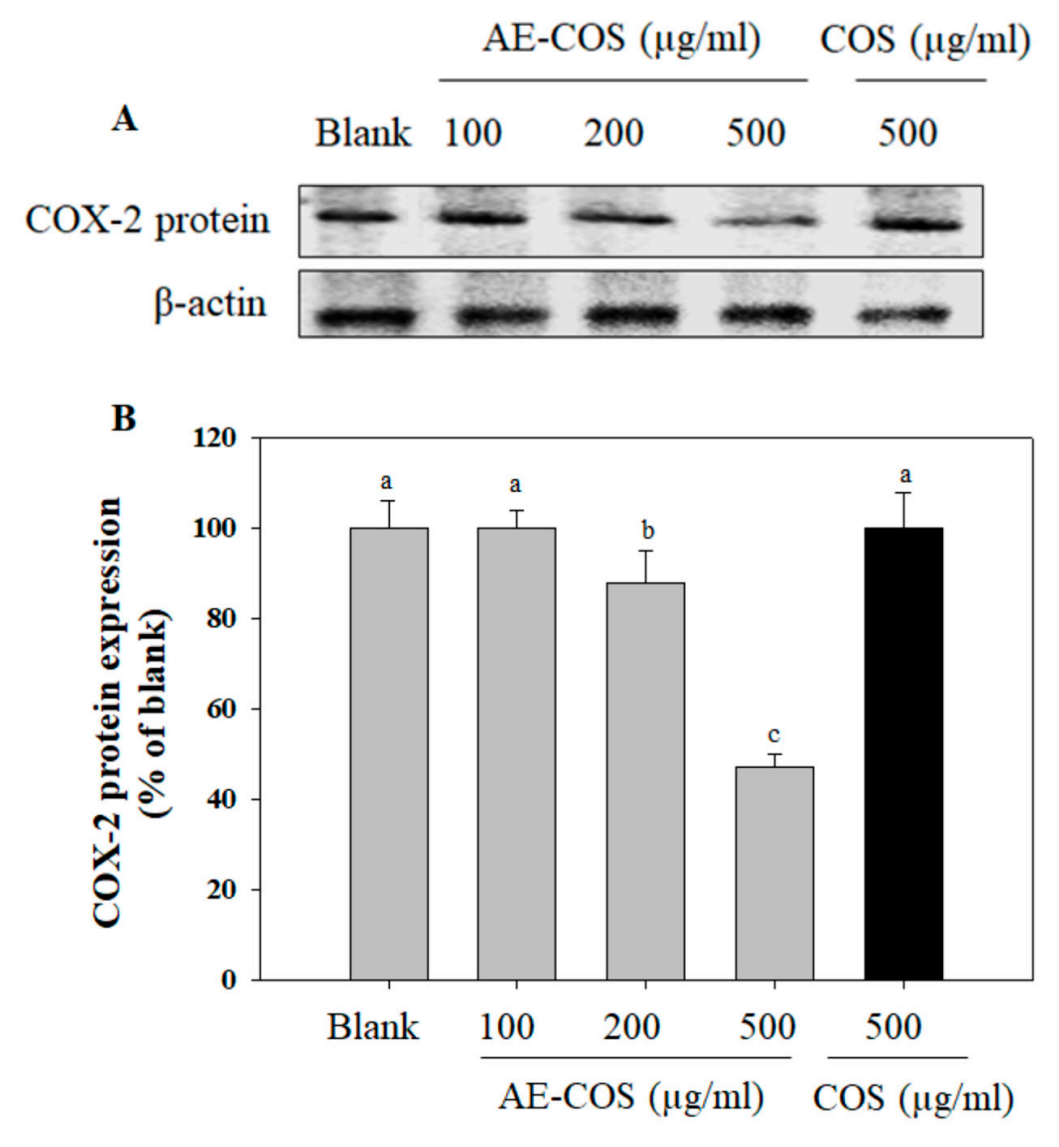
The suppressive effect of AE-COS on COX-2 protein expression in A549 cells. The cells were treated with various concentrations of AE-COS for 24 h. (**A**) The level of COX-2 protein expression was determined by Western blot analysis. The β-actin was used as internal control. Blank: No addition of AE-COS. (**B**) Densitometrically calculated expression level of COX-2 was given as a percentage compared to the blank group. Each determination was made in triplicate, and the data were expressed as means ± SD. If two groups have different letters, they are significantly different from each other at *p* < 0.05. Blank is absent AE-COS or COS.

It was revealed that overexpression of COX-2 was detected in lung cancers. COX-2 was suggested to play a vital role in different steps of cancer progression, by increasing proliferation, inhibiting apoptosis, and inducing metastasis formation [22]. Therefore, suppression of COX-2 expression may, in part, contribute to the inhibition of cancer progression. Herein, the effect of AE-COS on COX-2 protein expression was investigated in A549 cancer cells. It was found that AE-COS induced a dose-dependent suppression on COX-2 protein expression (Figure 2). The suppressive effect was clearly observed at the concentration of 500 µg/mL as compared with the blank group. Notably, the suppressive activity of AE-COS on COX-2 expression was stronger than that of COS. Indeed, several COX-2 specific inhibitors such as SC-236 and celecoxib were determined to be able to inhibit cancer cell proliferation [23,24]. Therefore, the suppression of AE-COS on COX-2 expression may be suggested to cause the inhibition of A549 cancer cell proliferation.

### 3.3. Effect of AE-COS on Apoptotic Signailing Molecules

**Figure 3:**
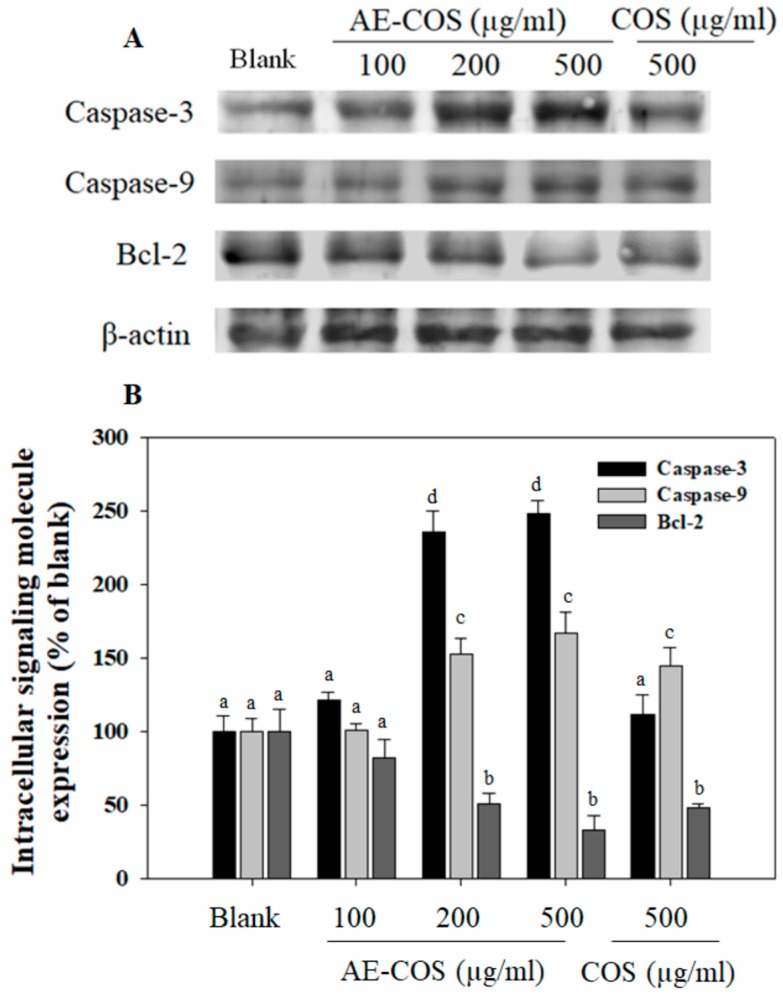
The effect of AE-COS on A549 cell apoptosis. The cells were treated with various concentrations of AE-COS for 24 h. (**A**) The expression levels of apoptotic signaling molecules were determined by Western blot analysis. The β-actin was used as internal control. Blank: No addition of AE-COS. (**B**) Densitometrically calculated expression level of signaling molecules was given as a percentage compared to the blank group. Each determination was made in triplicate, and the data were expressed as means ± SD. If two groups have different letters, they are significantly different from each other at *p* < 0.05. Blank is absent AE-COS or COS.

It was evidenced that Bcl-2 protein overexpresses in various cancer cell types, leading to the increase in the migratory and invasive potentials of cancer cells. Bcl-2 supports cellular viability and inhibits apoptosis in numerous cancer cells via inactivating caspase-9 and caspase-3 [25,26]. Thus, the down-regulation of Bcl-2 activation is considered as a potential target for anticancer therapy. Herein, the A549 cells were treated with different concentrations of AE-COS, and the levels of caspase-9, caspase-3, and Bcl-2 expression were analyzed by Western blot. Interestingly, AE-COS caused down-regulation of Bcl-2 expression in a dose-dependent manner (Figure 3). As the result, the expression levels of caspase-9 and caspase-3 were subsequently up-regulated in A549 cells after 24 h of AE-COS treatment. The down-regulation of Bcl-2 expression and the up-regulation of caspase-9 and caspase-3 by AE-COS treatment are the major effectors that may induce cell apoptosis and death as shown in Figure 1. The induction of cell apoptosis by AE-COS was observed to be more effective than that of COS at the same concentration in A549 cells. In the study of Karagozlu and colleagues, amino-derivatized COS also induced apoptosis via decreasing Bcl-2 expression in AGS cells [21]. Similarly, numerous natural products have been revealed to be potential for anticancer therapy due to inducing apoptosis in lung cancer, breast cancer, colorectal cancer, gastric cancer, prostate cancer, and liver cancer [27]. 

## 4. Conclusions

Numerous agents of natural or synthetic origin are common alternatives for cancer prevention and treatment in many countries around the world. In this study, the grafting of the aminoethyl group onto COS increased its anticancer activity in human lung cancer A549 cells. The anticancer activity of AE-COS was determined due to inhibiting cell proliferation and inducing cell apoptosis via down-regulation of Bcl-2 and up-regulation of caspase-3 and -9. Importantly, additional evaluation of the therapeutic effects of AE-COS on lung cancer would be positive evidence for its application as chemotherapy in cancer treatment in the future.
